# Large Language Model-Assisted Point-in-Time Interpretation of Advanced Hemodynamics in Liver Transplant Recipients: A Pilot Evaluation of Content Quality and Safety

**DOI:** 10.3390/jcm15020716

**Published:** 2026-01-15

**Authors:** Selma Kahyaoglu, Abdullah Kaygisiz, Izzet Alatli, Ayse Isik Boyaci, Emre Aray, Serkan Tulgar, Deniz Balci

**Affiliations:** 1 Department of Anesthesiology and Reanimation, Faculty of Medicine, Bahcesehir University, Istanbul 34732, Türkiye; selma.basyigit@bau.edu.tr (S.K.); serkantulgar.md@gmail.com (S.T.); 2 Department of Anesthesiology and Reanimation, Goztepe Medicalpark Hospital, E5 Uzeri Merdivenköy, 23 Nisan Sokak No. 17, Istanbul 34732, Türkiye; izzet.alatli@ou.bau.edu.tr (I.A.); ayse.isikboyci@ou.bau.edu.tr (A.I.B.); 3 Department of Gastroenterology Surgery, Faculty of Medicine, Bahcesehir University, Istanbul 34732, Türkiye; emre.aray@bau.edu.tr; 4 Department of General Surgery and Organ Transplantation, Faculty of Medicine, Bahcesehir University, Istanbul 34732, Türkiye; deniz.balci@med.bau.edu.tr

**Keywords:** large language models, ChatGPT, liver transplantation, PiCCO, hemodynamic monitoring, intraoperative decision-making, ARQuAT

## Abstract

**Background:** Large language models (LLMs) are increasingly used in clinical medicine, yet their ability to interpret advanced intraoperative hemodynamic monitoring—particularly in the context of liver transplantation—remains largely unexplored. In this proof-of-concept study, we evaluated ChatGPT’s capacity to interpret multimodal hemodynamic data derived from both standard anesthesia monitoring and the PiCCO system. The study also employed a structured assessment instrument (ARQuAT), adapted through a Delphi-based process to evaluate LLM-generated clinical interpretations. **Methods:** Ten key surgical–hemodynamic phases of liver transplantation were identified using a modified Delphi approach to capture the major physiological transitions of the procedure. Sequential screenshots representing these phases were obtained from five liver transplant recipients, yielding a total of 50 images. Each screenshot, along with standardized clinical background information, was submitted to ChatGPT. Five expert anesthesiologists independently assessed the model’s responses using the modified ARQuAT tool, which includes six content-quality domains (Accuracy, Up-to-dateness, Contextual Consistency, Clinical Usability, Trustworthiness, Clarity) and a separate catastrophic Risk item. Descriptive statistics were calculated for domain-level performance. Inter-rater reliability (Kendall’s W) and internal consistency (Cronbach’s alpha, McDonald’s omega) were also analyzed. All statistical analyses and visualizations were performed using NumIQO. **Results:** ChatGPT demonstrated consistently high performance across all content-quality domains, with median scores ranging from 4.6 to 4.8 and more than 90% of all ratings classified as satisfactory. Lower scores appeared only in a small subset of frames associated with abrupt hemodynamic changes and did not indicate a recurring weakness in any specific domain. Catastrophic Risk exhibited a pronounced floor effect, with 86% of ratings scored as 0 and only three isolated high-risk assessments across the dataset. Internal consistency of the six ARQuAT content domains was excellent, while inter-rater agreement was modest, reflecting ceiling effects and tied ratings among evaluators. **Conclusions:** ChatGPT generated clinically acceptable, contextually aligned interpretations of complex intraoperative hemodynamic data in liver transplant recipients, with minimal evidence of unsafe recommendations. These findings suggest preliminary promise for LLM-assisted interpretation of advanced monitoring, while underscoring the need for future studies involving larger datasets, dynamic physiological inputs, and expanded evaluator groups. The reliability characteristics observed also provide initial support for further refinement and broader validation of the Delphi-derived ARQuAT framework.

## 1. Introduction

Artificial intelligence refers to systems capable of mimicking human learning and human-like information processing, and in recent years it has been increasingly used across many fields to simplify and support human life [1]. Large language models -although often mistakenly used as a synonym for artificial intelligence- are in fact a subtype of machine learning built on deep-learning architectures, and they work by learning patterns from large volumes of text and generating new responses based on those patterns [2,3].

In medicine, artificial intelligence—particularly LLMs—is increasingly used across a wide range of tasks. These systems now assist clinicians in decision-making, establishing and refining differential diagnoses, patient triage, and interpreting laboratory, radiologic, pathology, or lesion-based images [1,4,5]. Their applications also extend to medical education and postgraduate training. As their presence has grown, the academic literature has rapidly expanded with studies aiming to assess their influence and practical value in these diverse settings [3,6,7]. In recent years, many large language models have also gained the ability to analyze images, and ChatGPT has become one of the most widely used platforms offering this feature [8]. Although several studies have explored the use of LLMs by providing patient information in text form or by pairing written data with clinical images, far fewer investigations have focused on presenting a direct screenshot of a physiological monitor and evaluating both the model’s ability to interpret the image and the quality of its subsequent clinical suggestions [9,10,11].

A growing number of anesthesia-focused studies have examined how LLMs perform in tasks ranging from blood gas interpretation to answering frequently asked questions about anesthesia practice [12,13,14]. However, their ability to interpret routine intraoperative monitoring or additional invasive modalities has not been adequately explored, and advanced hemodynamic systems have received virtually no attention [15,16]. In particular, no study to date has evaluated how an LLM handles the interpretation of complex parameters generated by devices such as the PiCCO system (Pulse Contour Cardiac Output). PiCCO monitoring provides minimally invasive hemodynamic assessment, enabling the measurement of cardiac output, preload, afterload, contractility, and the quantification of pulmonary edema [17,18]. This modality is widely used in critically ill patients and is often recommended in major surgical settings such as liver transplantation, where precise hemodynamic guidance is essential. These advanced devices should not be interpreted in isolation; rather, their measurements must be integrated with standard and other invasive monitoring tools for a comprehensive hemodynamic assessment.

Liver transplantation anesthesia differs substantially from routine anesthetic practice for both donor and recipient surgeries. The large number of parameters that must be tracked across distinct phases of the operation, many of which are multidimensional and rapidly changing, makes the anesthetic management particularly complex [19]. In addition, the need for advanced and sometimes unfamiliar perioperative monitoring adds another layer of difficulty. Because many training centers do not regularly perform liver transplantation, disparities in resident exposure can further limit hands-on experience [20]. As a result, the monitoring techniques used in transplant anesthesia require a high level of knowledge and experience, often achievable only after years of practice. The idea of using LLMs to assist with such demanding intraoperative monitoring tasks is therefore appealing; however, as with any emerging technology, their performance must be carefully evaluated by experts before these tools can be safely incorporated into routine clinical practice.

To address this gap, we designed a prospective, image-based evaluation in which ChatGPT was presented with sequential screenshots from liver transplant recipients monitored with both standard intraoperative devices and PiCCO-derived advanced hemodynamic parameters. To increase the diversity and representativeness of the dataset, screenshots were obtained from five patients across ten distinct intraoperative time points. Independent experts then assessed the model’s responses for content quality and safety, allowing a structured appraisal of how an LLM performs when confronted with high-complexity, point-in-time hemodynamic information. Importantly, this study was intentionally designed as a proof-of-concept investigation rather than an assessment of real-time clinical decision support.

## 2. Materials and Methods

### 2.1. Study Design and Ethics

This prospective evaluation study was conducted at Bahcesehir University School of Medicine to assess AI-generated, image-based interpretations produced by a large language model. Ethical approval was obtained from the university’s Non-Interventional Clinical Research Ethics Committee (Decision No. 2025-15/05; approval date: 1 October 2025), and all procedures were conducted in accordance with the principles of the Declaration of Helsinki. The study was designed to incorporate sequential, image-based inputs obtained from five liver transplant recipients at ten predefined intraoperative phases. These inputs consisted of screenshots capturing both standard monitoring and PiCCO-derived advanced hemodynamic parameters (PiCCO monitoring system; Pulsion Medical Systems SE, Feldkirchen, Germany), which were processed by a large language model and subsequently evaluated for content quality and safety by expert anesthesiologists using a structured assessment framework.

### 2.2. Definition of Intraoperative Phases and Construction of the Evaluation Tool

The intraoperative time points used in this study were determined by the authors using a modified Delphi process. Ten phases reflecting key hemodynamic and surgical milestones of liver transplantation were identified to capture the dynamic changes occurring throughout the procedure. These phases are summarized in Table 1.

The Assessment of Response Quality in AI-Generated Texts (ARQuAT) instrument was originally developed by Tulgar et al. [13]. For the present study, the tool was adapted and refined considering relevant literature [14]. The components of the modified ARQuAT framework are presented in Table 2.

### 2.3. Patient Selection and Imaging

The study included adult liver transplant recipients operated in October 2025. All adult patients undergoing elective transplantation during this period were eligible for inclusion. Exclusion criteria were: (1) emergency transplant procedures, (2) patients who arrived intubated from the intensive care unit, and (3) cases in which a screenshot could not be obtained, or the predefined phases could not be reliably identified. Images from such patients were excluded from the analysis. All images were captured across the predefined intraoperative phases by a single anesthesiologist (SK) and digitally archived without alteration.

### 2.4. Image Submission and LLM Interaction Workflow

Before initiating the interaction, the large language model received a standardized introductory prompt explaining that the study involved hemodynamic monitoring during liver transplantation and that ten predefined surgical–hemodynamic phases would be evaluated for each recipient. At the start of each case, the model was provided with non-evaluative background information, including the patient’s age, sex, height, weight, diagnosis, transplant indication, basic laboratory results, and MELD and Child-Pugh scores. These data were supplied solely to establish clinical context and were not themselves subject to evaluation or scoring. The full list of intraoperative phases—from post-induction stabilization to the early neo hepatic period—was then presented with their corresponding phase numbers, and the model was informed that subsequent screenshots would be labeled only by these phase numbers.

For each patient, screenshots of the anesthesia and PiCCO monitors, displaying PiCCO-derived parameters together with invasive arterial pressure, central venous pressure, heart rate, oxygen saturation, and ECG traces, were sequentially submitted to the paid, web-based version of ChatGPT (GPT-5.0; OpenAI, San Francisco, CA, USA) across the ten phases within a single continuous session between 3 and 5 November 2025. At each phase, the model was asked to provide scientific and clinically oriented interpretations, assessments, and suggestions based on the information visible in the images. It was explicitly instructed to maintain awareness of temporal continuity and, where appropriate, to compare the current phase with previous phases when formulating its interpretation. All outputs were collected verbatim and digitally archived for subsequent expert evaluation; no retrospective editing, correction, or manual adjustment of the model’s responses were performed.

For each patient, all screenshots and the corresponding ChatGPT-generated responses were compiled together and organized as separate, patient-specific files. Each phase’s image and its associated interpretation were paired and stored in sequence to preserve the temporal flow of intraoperative events. These files were then printed and assembled into structured physical folders prepared for distribution to the expert evaluators. The evaluation process was planned to be performed using these standardized physical folders to ensure consistency, prevent digital bias, and facilitate phase-by-phase scoring.

### 2.5. Selection of Evaluators and Evaluation Process

Five expert anesthesiologists specializing in liver transplantation were recruited through the Turkish Society of Anesthesiology and Reanimation (TARD) to serve as independent evaluator. These five experts were subsequently invited to independently review and score the model-generated interpretations using the modified ARQuAT tool. The expert anesthesiologists were aware that the interpretations were generated by an AI model (ChatGPT). The evaluations were conducted independently between 10 and 25 November 2025, using the finalized and archived AI-generated outputs.

### 2.6. Outcome Measurements

The primary outcome of the study was the performance of ChatGPT in interpreting intraoperative hemodynamic data from liver transplant recipients, assessed across the six ARQuAT content-quality domains: Accuracy, Up-to-dateness, Contextual Consistency, Clinical Usability, Trustworthiness, and Clarity. Domain performance was quantified using frame-level mean scores provided by five expert evaluators.

Secondary outcomes included:

1. Safety Assessment:
  Evaluation of ChatGPT’s risk profile using the ARQuAT Catastrophic Risk item, reported as the distribution of scores across all frames.
2. Inter-Rater Reliability:
  Agreement among the five evaluators for each ARQuAT domain, measured using Kendall’s coefficient of concordance (W) with tie correction.
3. Internal Consistency of the ARQuAT Instrument:
  Psychometric evaluation of the six content-quality domains using Cronbach’s alpha and McDonald’s omega (ω), including item-level diagnostics.

These outcomes were selected to provide a structured, multidimensional assessment of both ChatGPT’s interpretive performance and the reliability characteristics of the evaluation tool used in this study.

### 2.7. Statistics

All statistical analyses were performed using frame-level scores generated by five anesthesiologists. Similar validation studies assessing the performance of large language models have typically employed 5–8 expert raters; we selected five evaluators because the study required subspecialty-level expertise in liver transplantation hemodynamics and advanced use of PiCCO-derived flow parameters, making recruitment of a larger panel impractical and potentially inconsistent with the high domain knowledge required.

Descriptive statistics were used to summarize ARQuAT content-quality scores across the 50 intraoperative frames. For each domain (Accuracy, Up-to-dateness, Contextual Consistency, Clinical Usability, Trustworthiness, and Clarity), frame-level mean values were calculated and aggregated across all raters. Domain-level results are reported as median, interquartile range (IQR), and minimum–maximum values, with the proportion of satisfactory ratings (scores 4–5) calculated from the total of 250 observations per domain. The Catastrophic Risk item, scored on a three-point ordinal scale (0–2), was analyzed separately because it represents a distinct safety construct rather than content quality; its distribution was summarized using median, IQR, and absolute frequency of each score category.

To assess inter-rater agreement, Kendall’s coefficient of concordance (W) was computed for each ARQuAT domain, with correction for ties due to the expected restriction in score variability. Kendall’s W values were interpreted in the context of the observed ceiling effect, in which ratings were highly concentrated at the upper end of the scale, a phenomenon known to suppress concordance coefficients even when practical agreement is high.

Internal consistency of the ARQuAT scale was evaluated using Cronbach’s alpha and McDonald’s omega (ω). These analyses were performed only for the six content-quality domains, as inclusion of the Catastrophic Risk item—due to its limited ordinal range and distinct conceptual structure—artificially decreased internal homogeneity. Item-level diagnostics, including “alpha if item deleted,” were examined to determine the contribution of each domain to the latent construct of overall content quality.

All statistical analyses, along with all visualizations—including Sankey diagrams—were generated using the online analytics platform NumIQO (https://numiqo.com/ (Accessed on 29 November 2025)). A two-sided *p* value < 0.05 was considered statistically significant where applicable.

## 3. Results

A total of 50 intraoperative hemodynamic frames from five liver transplant recipients were evaluated by five anesthesiologists using a seven-item assessment tool. Based on frame-level mean values, ChatGPT demonstrated consistently high performance across all six core ARQuAT content-quality domains. As summarized in Table 1, median scores ranged narrowly between 4.6 and 4.8, with tight interquartile ranges, indicating a pronounced ceiling effect. Specifically, the median (IQR; min–max) values were 4.6 (4.4–4.8; 3.8–5.0) for Accuracy, 4.8 (4.4–4.8; 4.0–5.0) for Up-to-dateness, 4.6 (4.25–4.8; 3.4–5.0) for Contextual Consistency, 4.7 (4.4–4.95; 3.8–5.0) for Clinical Usability, 4.6 (4.4–4.8; 3.6–5.0) for Trustworthiness, and 4.8 (4.6–5.0; 3.8–5.0) for Clarity.

Across the 250 individual ratings per domain, the proportion of satisfactory scores (4–5) exceeded 90% in all categories, with the highest proportions observed in Clarity (94.4%), Up-to-dateness (93.6%), Clinical Usability (92.8%), Accuracy (92.4%), and Contextual Consistency (92.0%), with Trustworthiness similarly high (90.8%).

No true content hallucinations—defined as the generation of clinically incorrect or fabricated hemodynamic information—were identified in the evaluated outputs. However, despite all frame and phase labels being explicitly provided in the initial prompt, ChatGPT incorrectly referenced or misnamed the frame or phase label in 3 isolated instances. Importantly, these labeling inconsistencies did not affect the clinical interpretation, which remained aligned with the underlying screenshots and hemodynamic data.

Lower ratings (<4) were uncommon but showed limited clustering. The highest concentration was observed in Patient 2—Frame 8, which accumulated 12 low ratings distributed across all six content-quality domains, representing the most challenging frame for ChatGPT. Additional clusters were identified across multiple Frame 7 instances—specifically Patient 1—Frame 7, Patient 2—Frame 7, and Patient 5—Frame 7—each receiving 7–9 low ratings, primarily in Trustworthiness, Clinical Usability, and Contextual Consistency. A smaller cluster was also noted in Patient 1—Frame 9, predominantly reflecting lower Contextual Consistency scores. Outside these isolated clusters, low ratings were scattered, and no domain-specific or patient-specific pattern of underperformance was observed.

**Figure 1:**
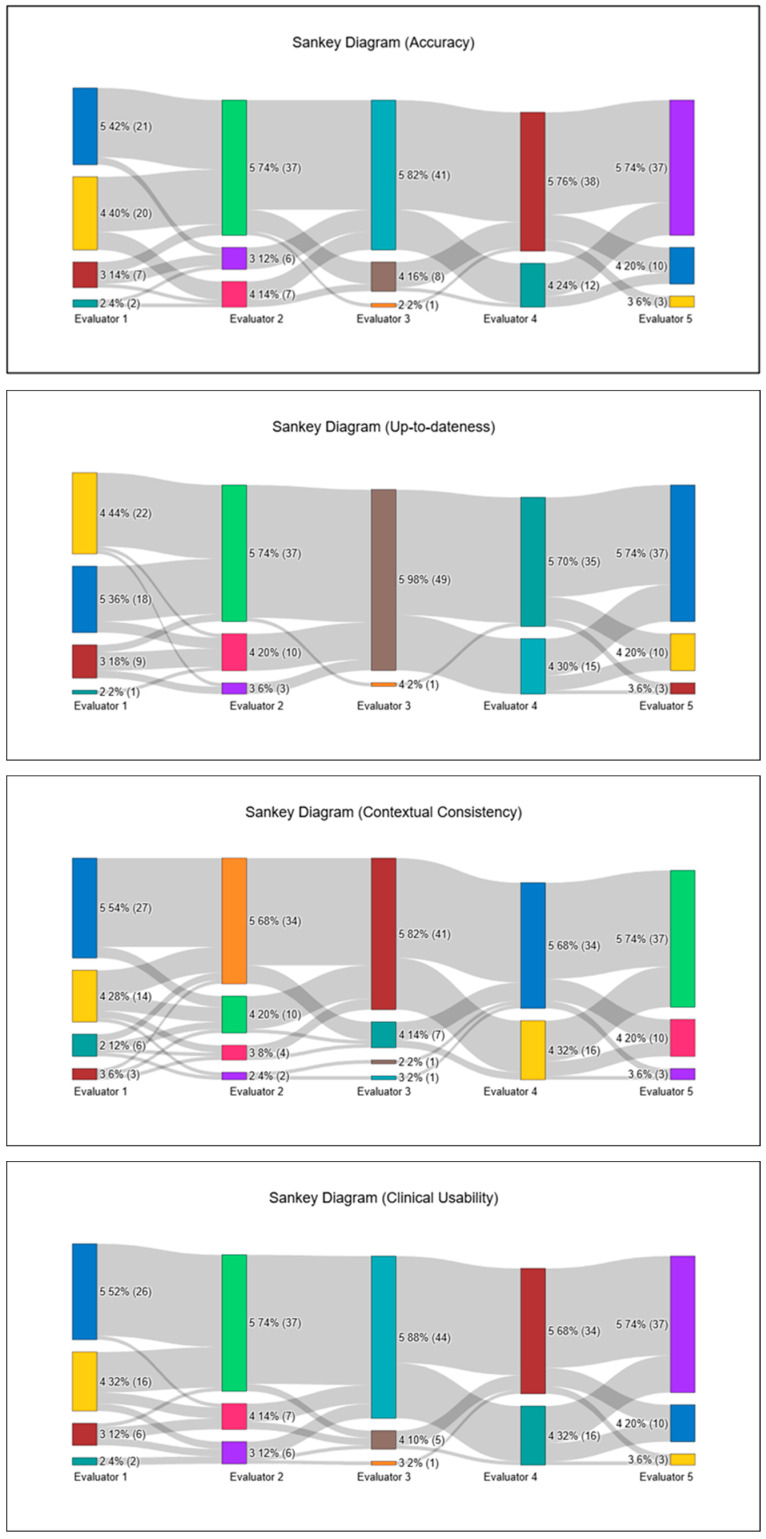

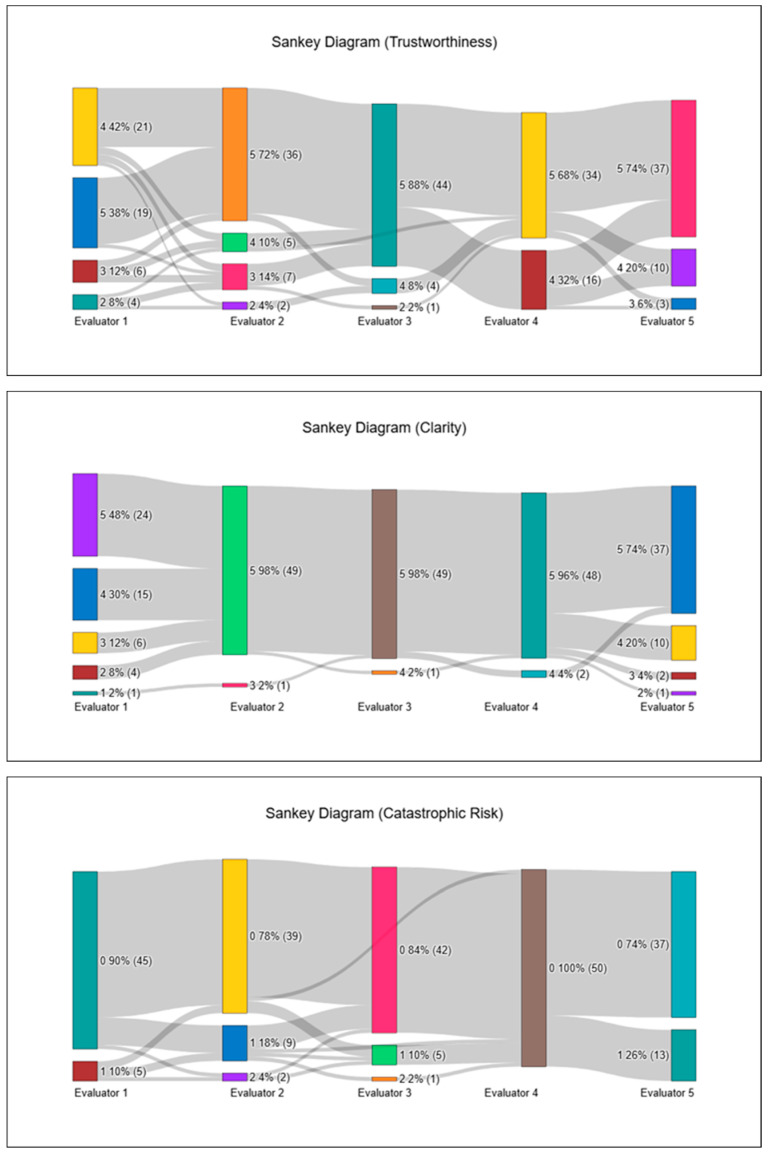
Sankey diagrams illustrating evaluator scoring patterns across all ARQuAT content-quality domains and catastrophic-risk assessments.

In this safety assessment, a Catastrophic Risk score of 0 indicates a clinically correct and risk-free interpretation, a score of 1 reflects minor issues requiring cautious clinical judgment, and a score of 2 denotes a clear patient-safety concern. The Catastrophic Risk item, scored separately on a three-point scale, showed a median of 0.0 (0.0–0.2; 0.0–0.8). Catastrophic Risk scores demonstrated a pronounced floor effect, with 215 of 250 ratings (86.0%) assigned a score of 0, indicating that the vast majority of ChatGPT-generated interpretations were perceived as entirely free of potentially harmful recommendations. Mild concern (score 1) appeared in 32 ratings (12.8%), but these were widely scattered across different patients and frames, with no evidence of clustering or concentration in any specific hemodynamic scenario. The highest risk category (score 2) occurred only 3 times (1.2%), each in a different patient—Patient 2/Frame 7, Patient 4/Frame 2, and Patient 5/Frame 4—and all were contributed by two evaluators (Evaluator 2 twice, Evaluator 3 once). Even in these cases, the remaining evaluators assigned 0 or 1, keeping the frame-level median at 0.0 throughout. Overall, this distribution confirms that ChatGPT did not produce any consistent or repeated high-risk interpretations, and that catastrophic-risk signals were both rare and non-focal. Overall, ChatGPT produced hemodynamic interpretations that were rated as clinically acceptable or better in the vast majority of intraoperative phases, with only a small number of frames demonstrating localized reductions in clarity, clinical usability, or contextual alignment. To illustrate inter-evaluator scoring patterns, all ARQuAT content-domain ratings and catastrophic-risk assessments are visualized as Sankey diagrams in Figure 1.

In addition to the aggregated domain-level results, the frame-level examination showed that the model’s performance was not uniform across all intraoperative moments. The lowest overall content-quality scores (mean: 3.93) occurred in Patient 2, Frame 8, which had the lowest mean ratings across the six ARQuAT content domains. This frame also generated the highest number of safety concerns, with three evaluators assigning non-zero catastrophic-risk scores (two scoring 1 and one scoring 2). Although such findings were rare in our dataset, this cluster of lower scores likely corresponds to the substantial physiological instability present at that time point. All evaluator ratings for this frame are presented in the Supplementary Materials to allow detailed examination.

In contrast, several frames received uniformly excellent evaluations. As an example, Patient 4, Frame 4, where every evaluator assigned a score of 5 across all content domains and 0 for catastrophic risk, is provided in the Supplementary Materials as a representation of peak model performance. Taken together, these two examples illustrate the range of interpretive behavior observed in our study and add nuance to the aggregate statistical results.

### 3.1. Internal Consistency of the ARQuAT

To evaluate the internal consistency of the ARQuAT instrument, psychometric analyses were performed across its seven scoring domains. Because the Catastrophic Risk item employs a different response structure (three-point ordinal scale) and conceptually represents a distinct safety construct rather than content quality, it was analyzed separately and not included in the core reliability calculation. The core ARQuAT scale, comprising the following six items—Accuracy, Up-to-dateness, Contextual Consistency, Clinical Usefulness, Trustworthiness, Clarity—demonstrated excellent internal consistency.

The six-item scale yielded a Cronbach’s alpha of 0.9585, indicating outstanding homogeneity among items. Complementarily, McDonald’s Omega (ω = 0.96) confirmed a strong unidimensional latent structure, further supporting the internal coherence of the scale. Item-level diagnostics showed that removal of any individual item did not increase Cronbach’s alpha, demonstrating that each item contributed positively to the underlying construct being measured.

The Catastrophic Risk item was retained as an independent safety indicator and reported separately. Its limited three-point distribution and highly restricted variance reduced its compatibility with the six content-quality items and weakened the scale’s internal consistency (α decreased from 0.9585 to 0.866 when included). Therefore, analyzing it independently aligns both statistically and conceptually with best psychometric practice. Importantly, the few instances rated as catastrophic risk were not related to misinterpretation of the displayed hemodynamic monitor data itself. Instead, these ratings reflected model-generated treatment suggestions that, if followed, could have adversely affected the patient’s clinical course. Specifically, these included delayed or insufficient fluid resuscitation in clearly volume-responsive states, as well as recommendations for unnecessary or excessive inotropic support that were not aligned with the patient’s contemporaneous clinical context. Notably, none of these frames were unanimously classified as catastrophic by all five assessors, indicating that the perceived potential harm of these recommendations was not consistently agreed upon across evaluators.

Taken together, these findings support the validity, internal coherence, and structural integrity of the six-item ARQuAT core scale, while recognizing Catastrophic Risk as a distinct and clinically meaningful supplementary safety metric (Table 3).

### 3.2. Inter-Rater Reliability

Inter-rater reliability across the seven ARQuAT domains demonstrated weak but consistent statistical agreement, with Kendall’s W values (corrected for ties) ranging from 0.23 to 0.29. Agreement coefficients were W = 0.23 for Accuracy and Clarity, 0.27 for Up-to-dateness, 0.26 for Contextual Consistency and Catastrophic Risk, and 0.29 for both Clinical Usability and Trustworthiness. Although these coefficients fall within the lower range of concordance, they are best interpreted in the context of the rating distribution: evaluators assigned highly homogeneous scores, with an overwhelming proportion of ratings in the 4–5 range across all domains. This pronounced ceiling effect, combined with extensive tied ranks inherent to Likert-type scores, substantially suppresses Kendall’s W despite the evaluators demonstrating strong practical convergence in their assessments. Notably, greater score dispersion was observed in a limited number of frames characterized by abrupt hemodynamic transitions, whereas more stable intraoperative frames showed highly consistent ratings. However, no systematic or domain-specific disagreement pattern was identified between stable and unstable frames. Therefore, tie-corrected coefficients were reported, as they more accurately reflect inter-rater agreement under restricted score variability.

## 4. Discussion

In this study, we examined ChatGPT’s ability to interpret point-in-time intraoperative hemodynamic data by evaluating 50 frames obtained from five liver transplant recipients. Across all six ARQuAT content-quality domains, evaluators consistently assigned high scores, with median values clustered between 4.6 and 4.8 and more than 90% of all ratings falling within the satisfactory range. Lower scores appeared only in a small number of frames associated with abrupt hemodynamic changes and did not indicate any domain-specific pattern of weakness. Safety assessments mirrored this trend, as catastrophic-risk scores demonstrated a marked floor effect with only three isolated high-risk ratings across the full dataset, indicating that potentially patient-safety–threatening interpretations were rare and non-recurrent rather than systematic. Reliability analysis further supported these findings: the ARQuAT core scale demonstrated excellent internal consistency (Cronbach’s α = 0.96), and although inter-rater agreement was modest (Kendall’s W = 0.23–0.29), this was expected given the pronounced ceiling effect and extensive tied rankings inherent to the scoring distribution. Overall, these results suggest that ChatGPT was generally able to generate clinically acceptable and contextually aligned interpretations of complex hemodynamic patterns during liver transplantation, with only limited, frame-specific reductions in clarity or clinical usability. This pilot demonstrates that a general-purpose large language model can generate structured and largely safe descriptions of complex hemodynamic snapshots, supporting its potential use as an educational tool, a cognitive aid for trainee case review, and a foundational step toward the development of more specialized, integrated decision-support systems.

Over the past several years, the use of advanced language models in medicine has expanded steadily, including within anesthesiology. Most published work has examined relatively narrow tasks—for example, providing answers to frequently asked perioperative questions, estimating ASA classification, or offering basic interpretations of arterial blood gas results—and these studies generally describe acceptable or even strong performance across different languages and platforms [21,22,23,24]. Despite this growing interest, their application to more physiologically complex intraoperative scenarios remains very limited. In anesthetic management for liver transplantation, clinicians frequently rely on advanced hemodynamic monitors such as PiCCO, often integrating these data with conventional monitoring to guide critical decisions [25,26]. Yet, to our knowledge, no prior study in anesthesiology has evaluated whether a language model can interpret these combined data streams in a clinically meaningful way. Similar examples are scarce in other medical fields as well. This gap highlights the novelty of our work, which represents an early attempt to assess model performance in a setting that demands continuous interpretation of dense, rapidly changing physiological information.

Liver transplantation presents a uniquely demanding anesthetic environment, distinguished from most other surgical procedures by its rapidly shifting physiological stages and the need for continuous, nuanced hemodynamic interpretation [27,28,29,30]. The dissection phase, the anhepatic period, and reperfusion each generate distinct and often abrupt changes in preload, afterload, cardiac output, metabolic status, and microcirculatory dynamics. As a result, advanced monitoring—particularly multimodal systems such as PiCCO—plays a far more central role than in routine major surgery [31]. Despite this, the comprehensive use of such monitoring is only modestly represented in standard anesthesia training, and many centers have limited exposure to transplant cases. Consequently, it is natural that anesthesiologists, even experienced ones, may occasionally seek expert input when interpreting complex perioperative data in this setting. In this context, evaluating whether a large language model can assist with interpreting dense, fast-evolving intraoperative information is both clinically relevant and addresses a gap in the existing literature.

When considered collectively, our findings suggest that ChatGPT demonstrated generally acceptable performance in interpreting both basic intraoperative monitoring and more advanced hemodynamic parameters in liver transplant recipients—a population in whom moment-to-moment physiological assessment is essential due to the complexity and rapid transitions between surgical phases. Across domains such as accuracy, contextual consistency, clarity, and clinical usefulness, the model’s outputs were largely judged as satisfactory, and overtly unsafe recommendations were uncommon—an encouraging observation for an initial exploratory study in such a demanding clinical context. The pattern of evaluator ratings, together with the reliability measures obtained, also provides preliminary support for the internal coherence of our Delphi-derived assessment tool. Although further validation in larger and more diverse datasets is warranted, these results indicate that both the LLM’s interpretive capabilities and the proposed evaluation framework may hold potential value for future investigations involving high-complexity intraoperative environments such as liver transplantation.

The consistently high domain scores should be interpreted with some caution. Rather than indicating expert-level clinical reasoning, these findings likely reflect the model’s strength in generating clear, structured, and generally accurate descriptions of hemodynamic parameters. In high-risk clinical settings such as liver transplantation, the distinction between a “good” and an “excellent” interpretation may hinge on subtle contextual nuances that are not fully captured by descriptive performance alone. Accordingly, an “acceptable” rating in the present study should be understood as indicating the absence of major errors and overall clinical plausibility, rather than equivalence to the judgment of an experienced transplant anesthesiologist. It should also be noted that we did not perform a formal comparison against expert-authored reference interpretations for each frame, which limits conclusions regarding the extent to which LLM-generated outputs approximate true expert reasoning.

Ideally, a clinically integrated artificial intelligence system would continuously receive and analyze raw, high-resolution physiological data—including beat-to-beat arterial pressure waveforms, electrocardiography, respiratory and ventilatory parameters, bleeding estimates, urine output, laboratory values, and concurrent monitoring modalities—and provide dynamic, time-sensitive decision support. Such an approach could enable earlier warning signals and more proactive perioperative management. However, the present study was not designed to evaluate such a fully integrated real-time system. Instead, as a proof-of-concept investigation, our primary aim was to assess whether a large language model could accurately and safely interpret clinically relevant hemodynamic information as it is typically perceived by anesthesiologists at the bedside, namely through monitor displays at predefined intraoperative moments. Evaluating performance on standardized, point-in-time screenshots allows a controlled and reproducible assessment framework and represents a necessary initial step. The findings of this study therefore serve as a foundational reference to inform and guide future research exploring fully automated, continuous, and multimodal AI-driven hemodynamic decision-support systems [16].

This study has several limitations that should be acknowledged. First, although we increased the total number of images by evaluating 10 phases from each of 5 liver transplant recipients, a larger patient cohort would undoubtedly strengthen the generalizability of the findings. Second, the evaluation focused exclusively on static intraoperative screenshots. In real clinical anesthesia practice—particularly in liver transplantation—decision-making relies not only on instantaneous hemodynamic values but also on continuous trends and additional contextual information such as blood loss, urine output, temperature changes, surgical duration, fluid management, and perioperative laboratory results. A more comprehensive, data-rich model incorporating continuous and dynamic physiological variables may enable future investigations into whether LLMs could eventually contribute to real-time anesthetic decision support; however, such fully dynamic and integrated applications lie beyond the scope of the present proof-of-concept study and may be explored in subsequent work building on these preliminary findings. Moreover, because liver transplantation represents a highly specialized and physiologically extreme surgical context, the present findings cannot be extrapolated to broader perioperative or non-transplant populations and should be interpreted strictly within the confines of this specific clinical setting.

Another limitation concerns the number of evaluators. Although five expert raters were included—consistent with the majority of comparable studies in the LLM-evaluation literature—a larger panel could provide more robust estimates of inter-rater reliability. Additionally, the assessment instrument used in this study was adapted from a Delphi process and has not undergone formal external validation. While our internal statistical analyses suggested good reliability and coherence of the tool, broader validation across different populations, settings, and use-cases would be necessary before widespread adoption. Because the expert anesthesiologists were aware that the interpretations were generated by ChatGPT, the possibility of conscious or unconscious evaluation bias cannot be excluded. In contrast to task-specific, large-scale models such as ECG-GPT [16], which are trained on millions of curated signals for a narrowly defined purpose, the present study deliberately focused on a general-purpose LLM to explore feasibility, safety, and evaluation methodology within a proof-of-concept framework rather than comparative benchmarking.

## 5. Conclusions

In this exploratory evaluation, ChatGPT demonstrated consistently high performance across all ARQuAT content-quality domains—Accuracy, Up-to-dateness, Contextual Consistency, Clinical Usability, Trustworthiness, and Clarity—with more than 90% of ratings judged satisfactory. Importantly, catastrophic-risk assessments revealed that unsafe recommendations were rare and non-repetitive, suggesting that the model generally operated within a clinically acceptable safety margin. Although inter-rater reliability was modest, the overall pattern of agreement, together with the strong internal consistency of the ARQuAT scale, indicates that the evaluative framework functioned coherently in this context.

These findings position our work as an early step toward understanding how large language models may support clinicians in interpreting complex intraoperative data in liver transplantation. Future studies incorporating larger datasets, continuous physiological trends, and broader clinical variables will be essential to determine whether such systems could eventually contribute to real-time anesthetic decision support or perioperative management.

## Figures and Tables

**Table 1:** Surgical–Hemodynamic Phases of Liver Transplantation.

Phase No.	Phase Name	Description
1	Post-Induction (Pre-dissection)	Early values obtained after PiCCO placement and stabilization of the patient’s hemodynamic response to intubation (approximately the 30th minute after intubation).
2	Beginning of Dissection	Initiation of liver mobilization with the first hemodynamic impact of surgical stress.
3	During Dissection (active surgical stress + blood loss)	Approximately 45 min after the beginning of dissection.
4	End of Dissection/Preparation of Portal Structures	Approach to the hepatic veins and porta hepatis, but no clamping yet.
5	Beginning of the Anhepatic Phase (immediately after liver removal)	The vena cava and portal vein were clamped.
6	Mid-Anhepatic Phase	This time point is determined based on the clinical estimation of the surgical/anesthesia team.
7	End of Anhepatic Phase/Preparation for Reperfusion	Immediately before completion of the anastomoses.
8	Moment of Reperfusion	The new liver graft was connected to the portal vein and hepatic vein.
9	Early Post-Reperfusion Stabilization (after 10 min)	First 10 min stabilization phase following reperfusion.
10	Neo-hepatic Phase	Immediately after intraoperative radiology confirmed arterial inflow.

**Table 2:** Components of the Modified ARQuAT Evaluation Tool.

A. Content Quality Items (Likert Scale: 1 = Very poor, 5 = Very strong)		
**Item**	**Assessment Question**	**Scale**
Accuracy	How well does the response align with the patient’s data and underlying pathophysiology?	1-2-3-4-5
Up-to-dateness	Is the response consistent with current knowledge, literature, or guidelines?	1-2-3-4-5
Contextual Consistency	How well does the answer fit the surgical phase and clinical context?	1-2-3-4-5
Clinical Usefulness	Does the response contribute meaningfully to clinical decision-making?	1-2-3-4-5
Trustworthiness	Does the output give the impression that the system would provide consistent answers in similar scenarios?	1-2-3-4-5
Clarity	Is the response clear, understandable, and easy to interpret?	1-2-3-4-5
B. Catastrophic Risk (Safety Assessment)		
**Catastrophic Risk**	**Description**	**Scale**
Catastrophic Risk	Does the response contain a serious error that could endanger patient safety if applied?	0 = Safe—Clinically correct and risk-free 1 = Needs Caution—Minor issue requiring careful interpretation 2 = Dangerous—Clear clinical error posing a patient-safety risk

**Table 3:** ARQuAT Domain Scores Based on Frame-Level Mean Ratings.

Metric	Median [IQR]	Range
Accuracy	4.6 [4.4–4.8]	3.8–5.0
Up-to-dateness	4.8 [4.4–4.8]	4.0–5.0
Contextual Consistency	4.6 [4.25–4.8]	3.4–5.0
Clinical Usability	4.7 [4.4–4.95]	3.8–5.0
Trustworthiness	4.6 [4.4–4.8]	3.6–5.0
Clarity	4.8 [4.6–5.0]	3.8–5.0
Catastrophic Risk	0.0 [0.0–0.2]	0.0–0.8

## Data Availability

The data presented in this study are not publicly available due to privacy and ethical considerations. Data may be made available from the corresponding author upon reasonable request.

## Supplementary Materials

The following supporting information can be downloaded at: https://www.mdpi.com/article/10.3390/jcm15020716/s1, Figure S1: Intraoperative monitoring screenshots, LLM-generated interpretation, and individual ARQuAT domain ratings for Patient 2, Frame 8 (lowest overall content-quality score); Figure S2: Intraoperative monitoring screenshots, LLM-generated interpretation, and individual ARQuAT domain ratings for Patient 4, Frame 4 (highest overall content-quality score).

## Abbreviations

The following abbreviations are used in this manuscript:

LLM	Large Language Models
MELD	Model For End-Stage Liver Disease
PiCCO	Pulse Contour Cardiac Output
ECG	Electrocardiogram
TARD	Turkish Society of Anesthesiology and Reanimation
IQR	Interquartile Range
