# Supplementary material for: Large Language Model-Assisted Point-in-Time Interpretation of Advanced Hemodynamics in Liver Transplant Recipients: A Pilot Evaluation of Content Quality and Safety
Source: J Clin Med. 2026 Jan 15;15(2):716. doi: 10.3390/jcm15020716 (PMC12841667; doi:10.3390/jcm15020716)
Supplement: Supplementary file 1 [file jcm-15-00716-s001.zip › jcm-4071305-supplementary.pdf]

# Supplementary Material

## Frame-Level Analysis of Extreme Content-Quality Scores

In addition to the aggregated domain-level analyses presented in the main manuscript, a detailed frame-level examination was performed to explore variability in the model's performance across distinct intraoperative moments. This supplementary analysis focuses on frames representing the lowest and highest overall content-quality scores observed in the dataset, thereby illustrating the range of interpretive behavior exhibited by the model.

The lowest overall content-quality ratings were observed in Patient 2, Frame 8, which yielded a mean score of 3.93 across the six ARQuAT content domains. This frame also generated the highest number of safety-related concerns in the study, with three independent evaluators assigning non-zero catastrophic-risk scores (two evaluators assigning a score of 1 and one assigning a score of 2). Although such safety signals were infrequent across the full dataset, their clustering in this specific frame suggests a relationship with the pronounced physiological instability present at that intraoperative time point. To enable transparent assessment, the corresponding intraoperative screenshot, the full large language model–generated interpretation, and all individual evaluator ratings for this frame are provided in the Supplementary Material.

In contrast, several frames demonstrated uniformly excellent performance. As a representative example, Patient 4, Frame 4 received the maximum score of 5 across all six ARQuAT content domains from every evaluator, with catastrophic-risk scores consistently rated as 0. This frame is included in the Supplementary Material together with the associated monitoring screenshot and model-generated interpretation as an illustration of peak model performance under stable physiological conditions.

Taken together, these contrasting examples highlight the contextual sensitivity of LLM-generated hemodynamic interpretations and provide qualitative depth beyond the aggregate statistical findings. By presenting both the lowest- and highest-scoring frames in full detail, the Supplementary Material allows readers to independently examine how physiological complexity and instability may influence model output quality and perceived safety.

**Supplementary Figure S1:** Intraoperative monitoring screenshots, LLM-generated interpretation, and individual ARQuAT domain ratings for Patient 2, Frame 8 (lowest overall content-quality score).

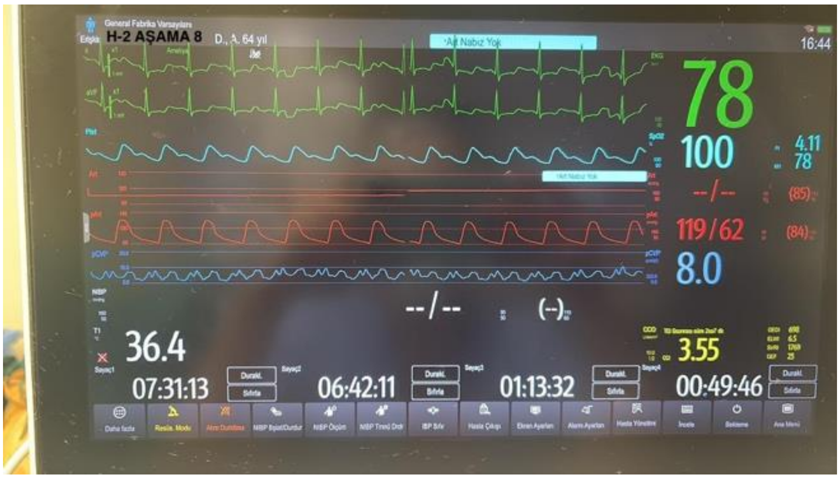


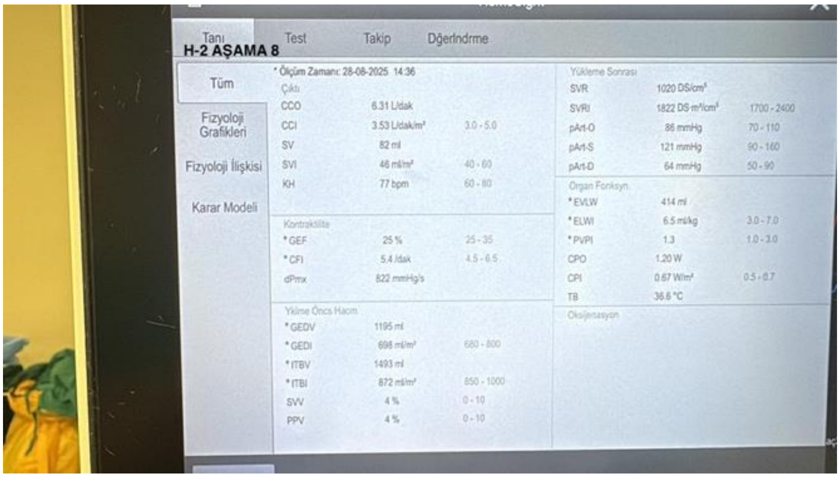


Answer of LLM Patient-2 Frame-8:

Phase 8 – End of Anhepatic Phase / Preparation for Reperfusion

General Status:
As the patient approaches reperfusion, the hemodynamic parameters appear relatively stable; however, several notable risk signals are present. Given that this stage represents preparation for one of the most critical periods of the procedure, close attention to preload, myocardial contractility, and systemic vascular resistance (SVR) dynamics is essential.

Monitoring Findings (BeneVision):

- HR: 78 bpm – within normal limits, with a downward trend compared to the previous phase.
- $SpO_2$: 100% – adequate oxygenation.
- Invasive Arterial Blood Pressure (pArt): 119/62 mmHg, MAP ≈ 81 mmHg – acceptable.
- CVP: 8.0 mmHg – decreased compared to earlier phases, indicating reduced preload.

- CCO: 3.55 L/min, CCI: 2.9 L/min/m² – within the low-normal range.
- Trend: A mild downward trend in cardiac index compared to previous phases is observed.

HemoSight Data:

- SV: 82 mL, SVI: 46 mL/m² – preserved relative to earlier phases.
- GEF: 25% – persistently low, indicating ongoing limitation in contractility.
- CFI: 5.4 /min – at the lower limit of normal.
- SVR / SVRI: 1020 $dyn \cdot s \cdot cm^{-5}$ / 1822 $dyn \cdot s \cdot m^2 \cdot cm^{-5}$ – low-normal; this profile may predispose to significant afterload fluctuations following reperfusion.
- EVLW / ELWI: 414 mL / 6.5 mL/kg – mildly elevated, suggesting a continued tendency toward pulmonary fluid overload.
- GEDI / ITBI: 698 mL/m² and 872 mL/m² – preload remains in the low-normal range.
- CPI: 0.67 W/m² – persistently low, indicating limited cardiac power reserve.

Clinical Interpretation:

- As the patient is prepared for reperfusion, preload is reduced and the cardiac index remains at the lower limit of normal.
- Myocardial contractility (GEF, CPI) is suboptimal and may further deteriorate during reperfusion syndrome.
- The risk of pulmonary fluid overload persists, as reflected by elevated ELWI.
- At this stage, both preload optimization (targeted fluid administration, albumin and/or plasma products) and readiness for inotropic and vasoactive support are critical.
- The risk of hypotension and low SVR during reperfusion is high, and the use of norepinephrine and/or vasopressin may become necessary.

Recommendations:

- Careful optimization of preload prior to reperfusion using a goal-directed approach while avoiding fluid overload.
- Consideration of inotropic support (e.g., dobutamine or milrinone), particularly if contractility further declines.
- Vasopressors should be readily available; a norepinephrine and/or vasopressin strategy should be considered to prevent reperfusion-associated hypotension.
- Close monitoring of ventilation and fluid balance to mitigate the risk of pulmonary edema.

**Supplementary Figure S2:** Intraoperative monitoring screenshots, LLM-generated

interpretation, and individual ARQuAT domain ratings for Patient 4, Frame 4 (highest overall content-quality score).

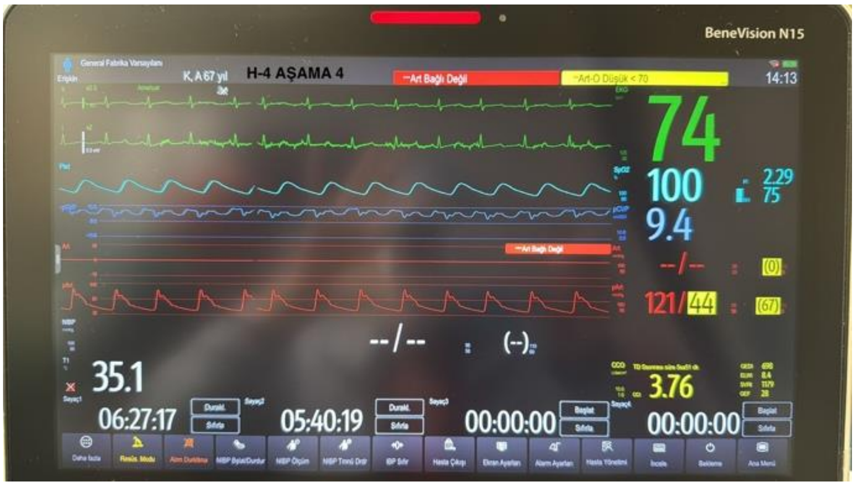


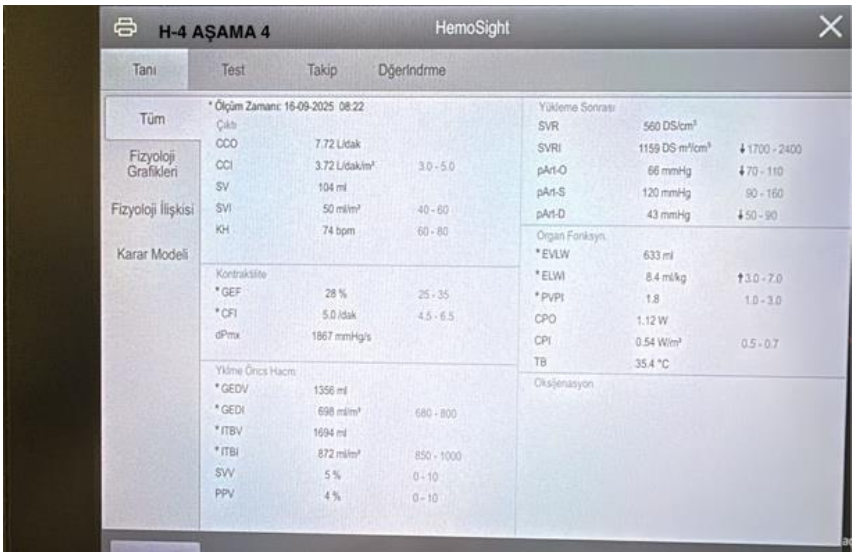


Answer of LLM Patient-4 Frame-4:

Phase 4 – End of Dissection / Preparation of Portal Structures

1. Heart Rate (HR)

- 74 bpm, decreased from 86 bpm in Phase 3.
- A calmer heart rate with reduced need for compensatory tachycardia, suggesting a balanced state in terms of preload and contractility.

2. Arterial Pressure (pArt-S / D / MAP)

- pArt-S: 120 mmHg, pArt-D: 43 mmHg, MAP ≈ 66 mmHg.
- Systolic pressure is preserved, whereas diastolic pressure is low.
- Reduced diastolic pressure is consistent with decreased afterload.

3. Cardiac Output / Index (CO / CI)

- CO: 7.72 L/min, CI: 3.72 L/min/m².
- Above normal and comparable to Phase 3 (7.56 / 3.65).
- Elevated CO in the presence of low afterload reflects a hyperdynamic circulatory state.

4. Stroke Volume / Index (SV / SVI)

- SV: 104 mL, SVI: 50 mL/m² – robust values.
- Consistent with the previous phase, supporting adequate preload.

5. Preload (GEDV, ITBV)

- GEDV: 1356 mL, GEDI: 698 mL/m² (near the lower limit).
- ITBV: 1694 mL, ITBI: 872 mL/m² – within normal ranges.
- Preload appears preserved, with no immediate need for additional volume.

6. Afterload (SVR / SVRI)

- SVR: 560 $dyn\cdot s\cdot cm^{-5}$, SVRI: 1159 $dyn\cdot s\cdot m^2\cdot cm^{-5}$ – markedly low (normal range: 1700–2400).
- Further reduced compared to Phase 3 (750 / 1552).
- Pronounced vasodilation is present, and pharmacologic support (e.g., vasopressors) may be required.

7. Contractility (GEF, CFI, dPmx)

- GEF: 28% (low-normal but stable).
- CFI: 5.0 /min – appropriate.
- dPmx: 1867 mmHg/s – adequate.
- Overall contractility is preserved, and additional inotropic support does not appear necessary.

8. Volume Responsiveness (SVV, PPV)

- SVV: 5%, PPV: 4% – low values.
- The patient is not volume responsive; further fluid loading is unlikely to be beneficial.

9. Pulmonary / Fluid Status (EVLW, ELWI, PVPI)

- EVLW: 633 mL, ELWI: 8.4 mL/kg (elevated; >7).
- PVPI: 1.8 – within normal limits.

- Interstitial pulmonary fluid accumulation is becoming more pronounced, warranting caution with fluid administration.

10. Organ Perfusion (CPO, CPI, Temperature)

- CPO: 1.12 W, CPI: 0.54 W/m² – at the lower limit of normal.
- Mild decline compared to earlier phases.
- Body temperature: 35.4 °C, indicating persistent mild hypothermia.

Overall Interpretation (Phase 4):

- A hyperdynamic circulatory state is present, characterized by elevated CO/CI and markedly reduced SVR/SVRI.
- Preload is adequate; however, afterload is significantly decreased, and vasopressor support may be required.
- Myocardial contractility is preserved.
- Pulmonary fluid burden continues to increase, as reflected by elevated ELWI.
- Organ perfusion power (CPO/CPI) remains borderline but is maintained through high cardiac output.

Therapeutic Priority:
Afterload support (e.g., norepinephrine), cautious fluid management due to elevated ELWI, and maintenance of overall hemodynamic stability.
